# Identification of a human mitochondrial isoform of TRPV1: a thermostat regulating mitochondrial maximal temperature?

**DOI:** 10.1007/s00018-026-06360-5

**Published:** 2026-07-23

**Authors:** Florian Beignon, Sylvie Ducreux, Artur Wardaszka, Joanna K. Bujak, Léa Tuifua, Camille Blaecke, Caroline Bosson, Lucie Maujean, Florentin Moulin, Yannick Le Dantec, Morgane LeMao, Adélie Mellinger, Méline Wery, David Goudenège, Arnaud Chevrollier, Salim Khiati, Hélène Tricoire-Leignel, Naig Gueguen, Nathalie Roux-Buisson, Julien Fauré, Piotr Bednarczyk, Guy Lenaers, César Mattei

**Affiliations:** 1 Univ Angers, MitoLab, Unité MITOVASC, UMR CNRS 6015, INSERM U1083, SFR ICAT, Angers, France; 2 University Claude Bernard Lyon1, CarMeN Laboratory-IRIS Team, INSERM, INRAE, Bron, 69500 France; 3 Department of Physics and Biophysics, Institute of Biology, Warsaw, University of Life Sciences - SGGW, Warsaw, Poland; 4 Univ. Grenoble Alpes, Inserm, U1216, CHU Grenoble Alpes, Grenoble Institut Neurosciences, Grenoble, 38000 France; 5 Univ Angers, SFR ICAT, Angers, France; 6 Univ Angers, CarMe, Unité MITOVASC, UMR CNRS 6015, INSERM U1083, SFR ICAT, Angers, France; 7 Service de Biochimie Et Biologie Moléculaire, CHU d’Angers, Angers, France; 8 Service de Neurologie, CHU d’Angers, Angers, France

**Keywords:** TRPV1, Mitochondria, Calcium, Thermoregulation, Mitochondrial targeting sequence, Ion channel

## Abstract

Mitochondria are well known to produce ATP for cell energy requirements and thermogenesis simultaneously by the respiratory chain, with a potential optimum temperature of 50 °C, while deleterious overheating should be prevented by a yet unknown system. Here, we report the identification of a human Transient Receptor Potential Vanilloid 1 (mitoTRPV1) alternative isoform located in the mitochondrial inner membrane, which acts as a thermostat regulating mitochondrial maximal temperature. The ubiquitously expressed mitoTRPV1 open reading frame overlaps *TRPV1* exons 1 and 2 and intron 2 in a + 1 frame, encoding for an effective 150 amino-acid N-terminal mitochondrial targeting sequence (MTS) conserved amongst mammalian species, followed by the 687 amino acids of the TRPV1 C-terminal sequence. Pharmacological activation of mitoTRPV1 induces a low cytoplasmic Ca^2+^ increase and mitochondrial cooling without modification of mitochondrial respiration and ATP production. We conclude that, in our experimental conditions, mitoTRPV1 acts as a thermostat to promote mitochondrial thermolysis without impairing respiration and ATP production. They point to the potential implication of mitoTRPV1 in human diseases related to temperature dysregulation, and should be further studied in physiological conditions in cellular and animal models expressing mitoTRPV1 endogenously.

**Supplementary Information:**

The online version contains supplementary material available at https://doi.org/10.1007/s00018-026-06360-5.

## Introduction

Mammals are homeothermic species that balance thermogenesis and thermolysis to adapt body temperature in changing environmental conditions, a critical process, as variations of plus or minus 5 °C lead to morbidity and lethality [1, 2]. Thermogenesis mainly relies on heat generated by the mitochondrial oxidation-phosphorylation process, promoting mitochondria as cell radiators [3]. In line with this concept, the mitochondrial temperature in cells can be up to 15 °C above that of their external environment, thus reaching about 50 °C [4–6], a temperature at which the enzymatic activities of respiratory chain complexes complex III and IV and F0F1-ATPase are supposed to be the highest [5]. However, this hypothesis is a matter of intense debate [7], as the mechanisms sensing and monitoring mitochondrial maximal temperature are unknown, thus limiting our understanding of mitochondrial thermogenesis control and its contribution to homeothermy in mammals. Nevertheless, the Ca^2+^ channel TRPV4 has been demonstrated to partially localize to the mitochondrial network, where it activates thermogenesis at temperature below 37 °C [8]. To gain novel insights in this topic, we hypothesized that mitochondria must include a thermo-sensor modulating maximal thermogenesis and preventing deleterious effects associated with hyperthermia.

Thermo-TRPs are Transient Receptor Potential (TRP) ion channels activated by heat (TRPV1-4, TRPM2-5) or by cold (TRPA1, TRPM8 and TRPC5) and act as thermo-sensors in the peripheral nervous system to detect environmental temperatures [9]. Most TRPs are localized at the plasma membrane or in intracellular organelles, including mitochondria, but none of them has ever been proposed to act as a thermostat limiting mitochondrial warming. TRPV1 is a polymodal cation channel activated by temperatures above 43 °C, by capsaicin (CAP)—the pungent “burning” component of red chili peppers—and by resiniferatoxin (RTX), its most specific agonist [10, 11]. TRPV1 is implicated in the noxious sensing of thermal and chemical stimuli, which activate somatosensory neurons responsible for acute and inflammatory pains [12]. Consequently, pharmacological TRPV1 activation in primary afferent sensory neurons lowers body temperature by promoting vasodilation and sweating [13, 14], while conversely, its inhibition displays analgesic actions and induces deleterious hyperthermia symptoms [15].

In line with these findings, *TRPV1* variants were associated with a predisposition to acute life-threatening hyperthermia, both in individuals facing exertional heat stroke and in patients treated with halogenated anaesthetics [16, 17]. In all cases, syndromes were associated with a failure to counteract hyperthermia with dramatic consequences on brain, muscle, liver and kidney functional integrity [18, 19]. Additional *TRPV1* variants abrogating TRPV1 activity or partial deletions of the *TRPV1* promoter were identified in patients presenting heat hyposensitivity, delayed response to noxious heat, inflammatory hyperalgesia and insensitivity to CAP [20, 21]. These clinical data demonstrate that TRPV1 is fundamental in responding to thermal and noxious stresses and adapting body temperature, while *Trpv1-*KO mouse models only illustrate altered responses to noxious stimuli without any thermoregulation defect [22]. Beyond these roles, alternative TRPV1 expression and localization have led to novel TRPV1 functions in both neuronal and non-neuronal cells [23]. Indeed, aside from its embedment in the plasma membrane, TRPV1 was also localized in intracellular compartments, as the sarcoplasmic reticulum membrane of skeletal muscle, acting as a Ca^2+^ leak channel [24]. It is also expressed in the mitochondria of cardiomyocytes, where it stimulates ATP synthesis [25], in microglial cells to promote cell migration and reactive oxygen species production [26, 27], and in endothelial cells contributing to diabetes onset [28]. Nevertheless, the role of TRPV1 in intracellular temperature sensing has never been reported.

Here, we describe and characterize a novel mammal-specific TRPV1 mRNA variant encoding an isoform located in the mitochondrial inner membrane (hereafter called mitoTRPV1) and its involvement in the control of mitochondrial temperature, comparatively to the conventional TRPV1 isoform located at the plasma membrane (hereafter called pmTRPV1). Our results show that mitoTRPV1, when expressed in a heterologous system, acts as a thermostat, cooling down the mitochondrial temperature, without affecting respiration and ATP production.

## Results

### mitoTRPV1 is encoded by a novel atypical ORF

**Fig. 1:**
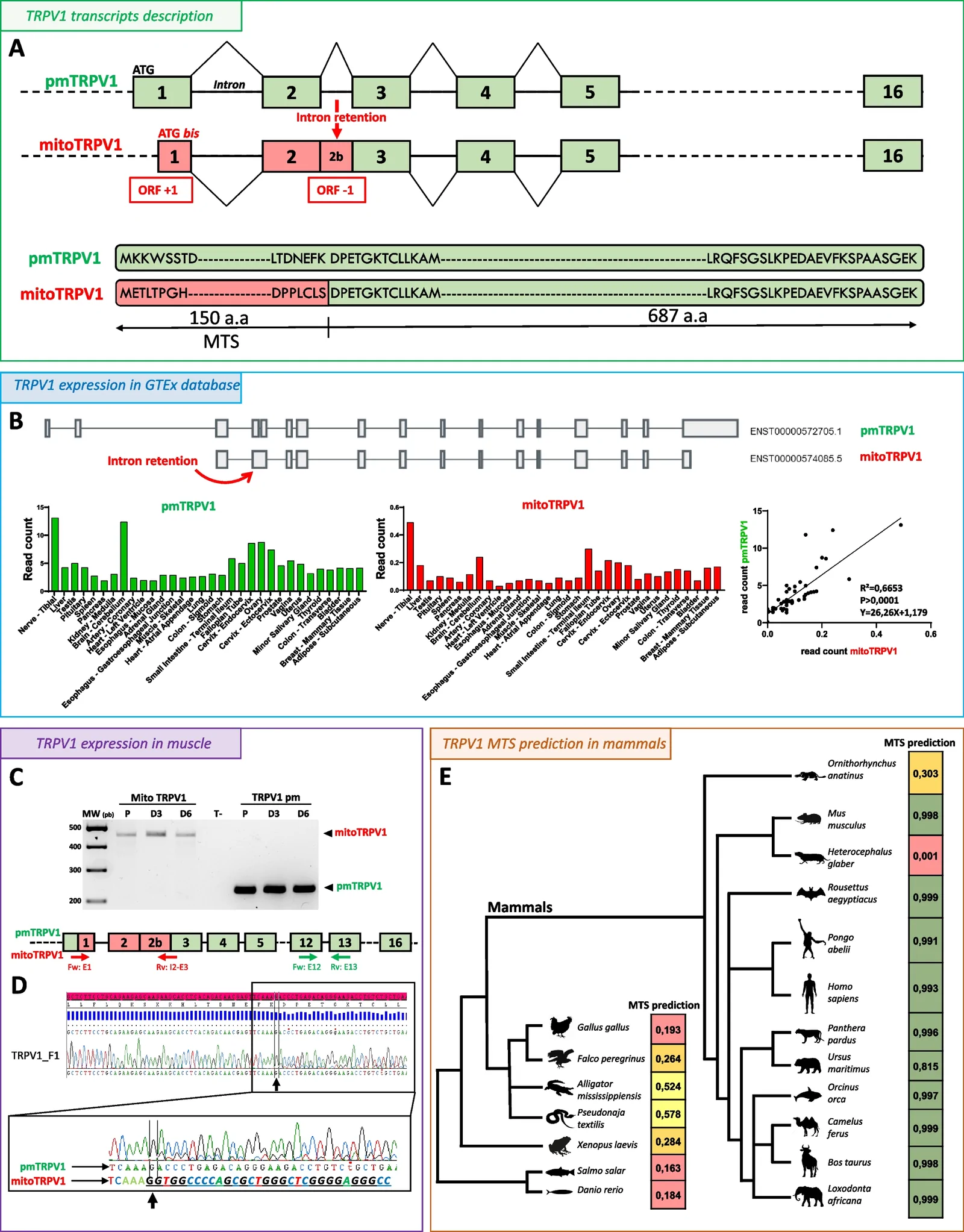
Identification of mitoTRPV1 cDNA, protein sequence and expression levels in human cells and tissues. **A** Open reading frame organization of pmTRPV1 and mitoTRPV1 variants. MitoTRPV1 ATG initiation codon lies in exon1 in a + 1 frame and generates an overlapping ORF encompassing also exon2 and the 70 nt long intron2, which retention restores the consensual TRPV1 ORF. Comparison of the protein structure of the two TRPV1 isoforms, the one located in the plasma membrane (pmTRPV1) on top and the one with a mitochondrial targeting sequence (MTS in pink, amino acids 1–150) (mitoTRPV1) on the bottom. **B** Structures of TRPV1 coding sequence of pmTRPV1 and mitoTRPV1 with intron 2 retention reported in the GTEx Portal database. Quantitative tissue expression of the pmTRPV1 (green) and mitoTRPV1 (red) mRNA variants in the GTEx Portal database. Ratio (bottom) between mitoTPRV1 and pmTRPV1 expression, inferred from the histograms and values from d. **C** The mitoTRPV1 transcript expression was detected by PCR on immortalized human cultured myoblast in proliferation (P) or after 3 (D3) and 6 (D6) days of differentiation into myotubes. PCR amplification was performed with two specific pairs of primers Fw:E1/Rv:I2-E3 for mitoTRPV1 and Fw:E12/Rv:E13 for pmTRPV1. PCR products are labeled pmTRPV1 for the full lenght isoform (225 bp) and mitoTRPV1 for the mitochondrial isoform (450 bp). (T-) = PCR control without DNA. **D** The mitoTRPV1 transcript is expressed in a human muscle biopsy. Sanger chromatogram of TRPV1 transcript sequenced with TRPV1-F1 primer is shown. The arrow indicates the position where 2 sequences are superimposed resulting from the co-expression of the two transcripts. The boxed area shows the superposition of the pmTRPV1 sequence with the mitoTRPV1 sequence (in italic) expressed in the background. **E** Phylogenetic and score prediction for the presence of an MTS in an alternate ORF in *TRPV1* gene from the major vertebrate species lineages, using the Mitoprot II software. Mammals are boxed in red

To identify a mitochondrial “hot” thermostat, we screened mRNA databases for TRP variants including a potential mitochondrial targeting sequence (MTS), using the MitoProt-II prediction software V1.101. This process identified a *TRPV1* cDNA (accession number DQ177332.1 (NCBI) and ENST00000574085.5 (Ensembl)) starting at an alternate ATG initiation codon in exon1, generating an overlapping + 1 ORF encompassing exon1 3’-end, the complete exon2, the 70 nt-long intron2, followed by *TRPV1* consensual coding frame from exon3 to exon16 (Fig. 1A and S1). Translation of the nucleotide sequence leads to a predicted 150 amino acid (aa) mitochondrial targeting sequence (MTS) sequence at the N-terminal domain (0.9968 probability according to MitoProt-II and a predicted cleavage site at position 138) followed by the 687 aa of TRPV1 C-terminal sequence. This isoform was called mitoTRPV1. Browsing the GTEx portal database (https://www.gtexportal.org/home/, accessed on 27/04/2026) confirmed the existence of *mitoTRPV1* mRNA with the intron2 retention between exon2 and 3. It further disclosed its ubiquitous expression in 32 human tissues, paralleling that of pmTRPV1 mRNA (R^2^ = 0.6653), although with an abundance 26 times lower than that of the conventional pmTRPV1 (Fig. 1B). To assess the expression of the mitoTRPV1 transcript in human tissue, we first used human immortalized muscle cells (myoblasts) from a healthy donor. Myoblasts were kept in proliferation or set to differentiation into multinucleated myotubes for 3 to 6 days. After RNA extraction and RT-PCR, the presence of the alternative splicing event leading to the mitoTRPV1 transcript was detected using a pair of primers encompassing the exon1-exon 2b junction (Fig. 1C). To investigate the level of expression of mitoTRPV1 further, we took advantage of genetic investigations performed on individuals addressed for an exertional heat stroke episode [17], for whom the Sanger sequencing of TRPV1 mRNA was performed from a muscle biopsy. The electrophoregram of exon 1 and 2 boundaries’ sequencing (Fig. 1D) disclosed a superposition of 2 sequences starting at the position corresponding to the intron 2 junction. The strongest signal corresponds to the sequence of pmTRPV1 and the underlying sequence to the sequence of intron 2 confirming that both isoforms are expressed in human skeletal muscle, although the sequence of mitoTRPV1 shows, as expected, a lower endogenous expression level. We next screened for the presence of a sequence encoding an MTS in the *TRPV1* genomic sequence from vertebrate animal species using the MitoProt-II program, considering all possible initiation codons and downstream ORF extensions. We evidenced that in virtually all mammal species, the probability of generating an MTS is close to 1, while this probability was absent in homeothermic birds, or poikilotherm reptiles, batracians and fishes (Fig. 1E). Interestingly, this probability was also low in the platypus (*Ornithorhynchus anatinus*), the Mongolian gerbil (*Meriones unguiculatus*) and the naked mole-rat (*Heterocephalus glaber*) (Fig. 1E, Fig. S2), species which display alternative or no thermoregulatory mechanisms. These data suggest that mitoTRPV1 may link functions related to placental development and homeothermy. They are also consistent with the phylogenetic tree inferred from TRPV1 alternate N-ter sequences (Fig. S2), where the Mongolian gerbil and the naked mole-rat show sequences closer to those of *Xenopus laevis* than to the rest of the mammalian clade.

### mitoTRPV1 is located in the mitochondrial inner membrane

**Fig. 2:**
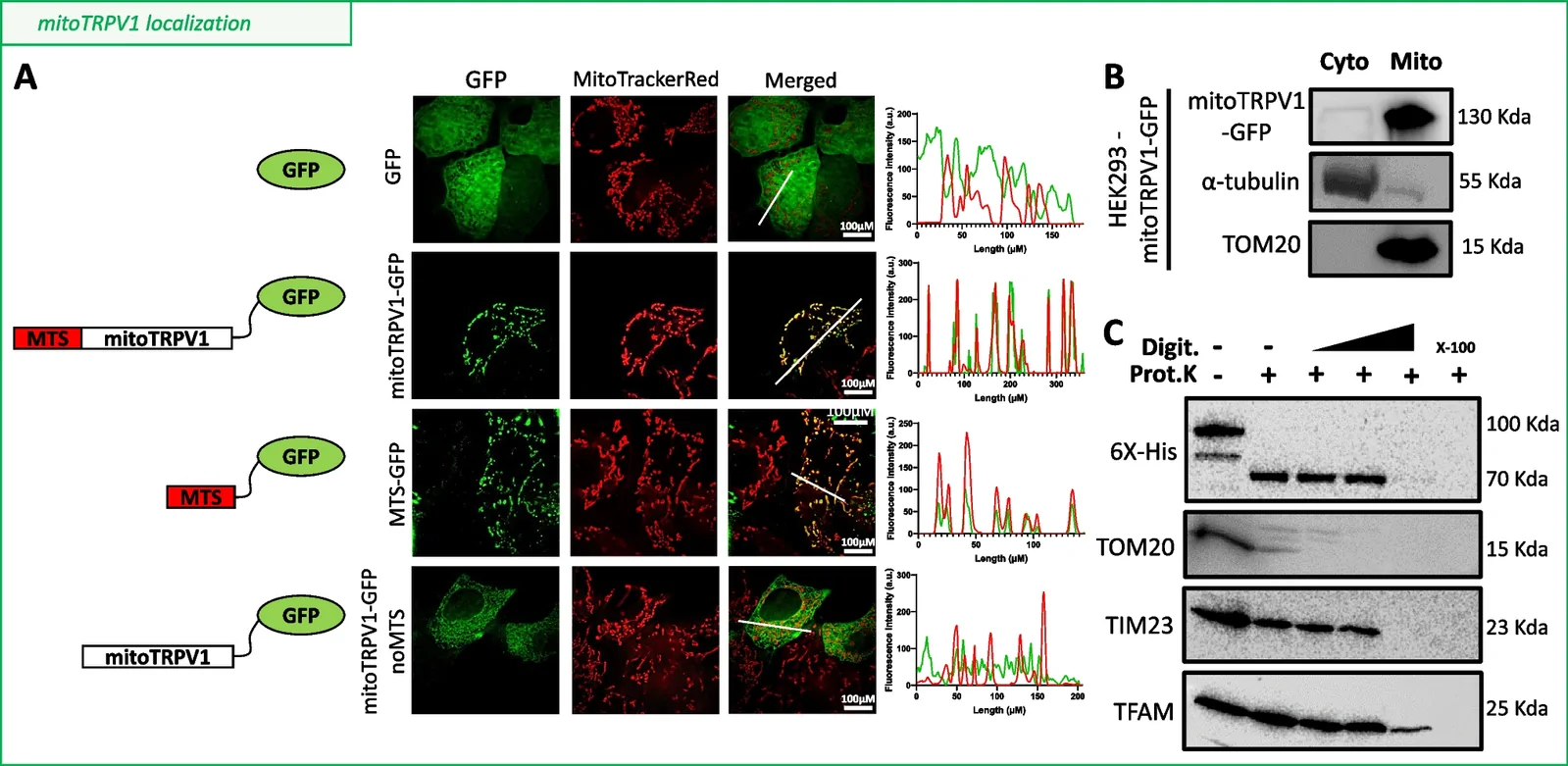
Mitochondrial localization of mitoTRPV1. **A** Co-localization analysis of GFP (green) and Mitotracker (red) fluorescence in MCF-7 cells expressing GFP alone (1st line), mitoTRPV1-GFP (2nd line), MTS-GFP (3rd line) and mitoTRPV1-GFP deleted from the MTS (4th line), together with the histogram transection levels of green and red fluorescences. **B** Western blot analysis of cytoplasmic (left) and mitochondrial (right) fractions of HEK293 cells expressing mitoTRPV1. **C** Proteinase K protection assay of a mitochondrial fraction from cells expressing a 6-His-tagged mitoTRPV1 isoform. Column 1 corresponds to the untreated mitochondrial fraction. Columns 2 to 6 show the fraction treated with proteinase K without digitonin (column 2), with increasing concentrations of digitonin (column 3–5), or with Triton X100 (column 6). Representative Western blots obtained with antibodies against 6-His-tagged TOM20, TIM23, and TFAM

To challenge the predictive in silico value of mitoTRPV1 MTS and its functional relevance, we cloned GFP fusion proteins, including the whole or parts of the mitoTRPV1 cDNA sequence, and expressed them in MCF7 cells. Compared to the Mitotracker fluorescence, expression of GFP alone or GFP fused to mitoTRPV1 deleted of its MTS sequence, displayed a cytoplasmic localization. Conversely, the expression of mitoTRPV1 or its MTS alone in fusion with the GFP displayed a mitochondrial fluorescence perfectly superposed to that of the Mitotracker (Fig. 2A). This result indicates that the mitoTRPV1 MTS is required and sufficient to drive TRPV1 to mitochondria, even if we cannot rule out that a small fraction of mitoTRPV1 could be in the ER membrane. The same results were observed for HEK293 cells expressing mitoTRPV1 (data not shown). Further Western blot analyses of a mitochondrial fraction of HEK293 cells expressing mitoTRPV1-GFP confirmed the mitochondrial localization of mitoTRPV1 (Fig. 2B). To address the mitochondrial sub-localization of mitoTRPV1, we first excluded that mitoTRPV1 is in MAMs (S3 Fig), then we performed a Proteinase-K protection assay on mitochondria purified from HEK293 cells expressing mitoTRPV1-6His. Without treatment, mitoTRPV1 appears as two bands, one corresponding to the full-length protein and the other to a shorter isoform, probably cleaved of its MTS (Fig. 2C, lane 1). Initial treatment with Proteinase-K in the absence of digitonin led to a second mitoTRPV1 cleavage, paralleling the degradation of TOM20 located at the outer membrane (Fig. 2C, lane 2), suggesting that either the N-ter (after the MTS cleavage) or the C-ter end of mitoTRPV1 lays in the intermembrane space (IMS). The addition of increasing digitonin concentrations led to a mitoTRPV1 protection pattern similar to the one of TIM23, suggesting that the core of the mitoTRPV1 protein is embedded in the mitochondrial inner membrane (Fig. 2C, lanes 3 to 6). This result is also supported by the “DeepMito” tool (http://busca.biocomp.unibo.it/deepmito/, accessed on 02/06/2025) that predicts mitoTRPV1 localization in the mitochondrial inner membrane with a score of 0.73. Thus, the presence of an MTS indicates that mitoTRPV1 is unequivocally a mitochondrial protein, with its core-channel domain embedded in the inner membrane.

### Electrophysiological characterization of mitoTRPV1

**Fig. 3:**
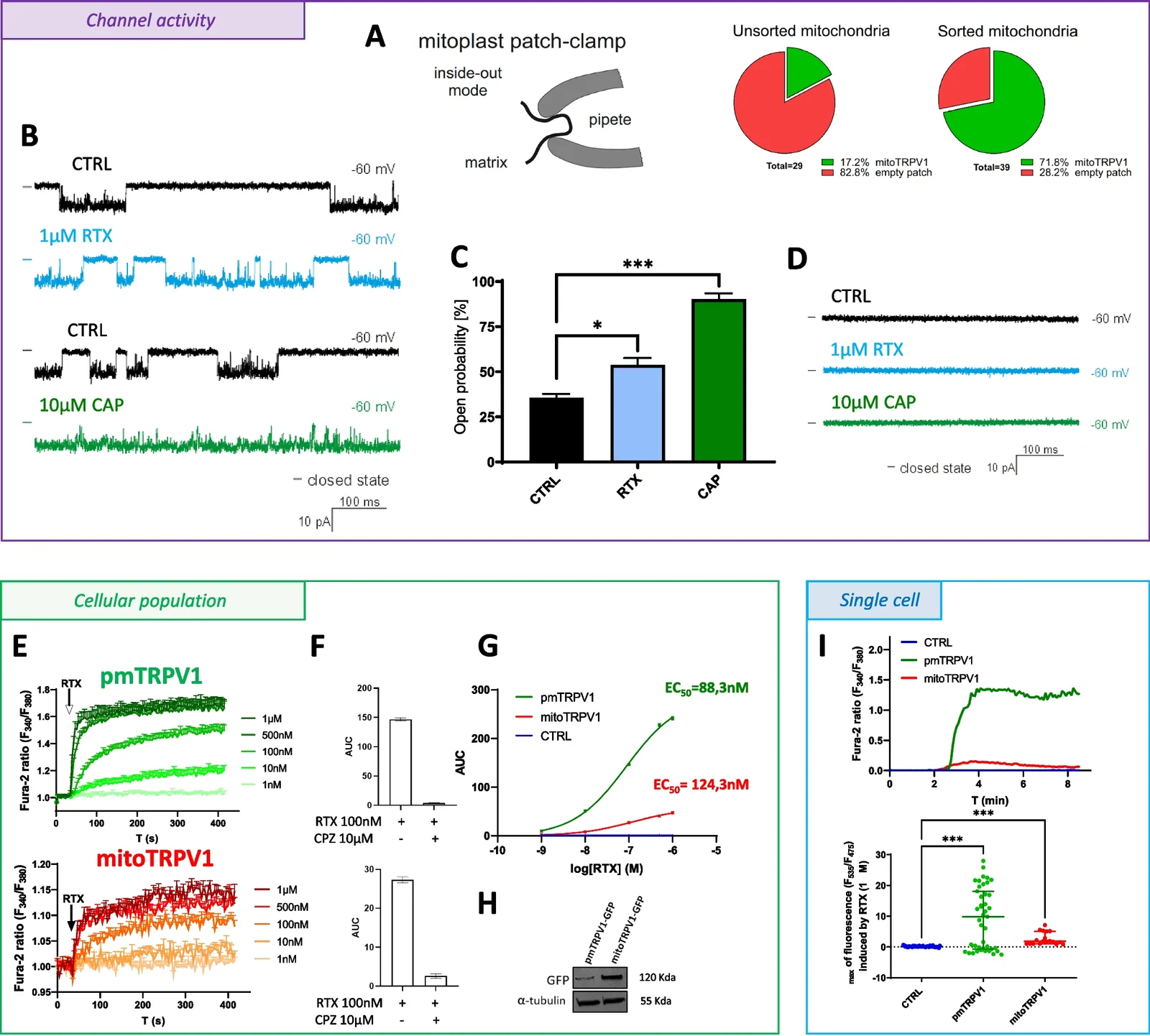
Assessment of mitoTRPV1 pharmacological properties in mitoplasts and involvement in Ca^2+^ homeostasis. **A** Diagram showing the patch-clamp configuration used during electrophysiological measurements. The pie chart displays the percentage of mitoTRPV1 channel currents (green) possessing a conductance of ∼157 pS relative to the total number of patch-clamp experiments performed using mitoplasts isolated from transfected HEK293 cells – unsorted mitochondria (left side), sorted mitochondria (right side). Red segments represent a percentage of patches without detectable channel activity (”empty patches”). The chart highlights the efficiency of the mitochondrial sorting process by illustrating the increased detection rate of mitoTRPV1 channel activity in sorted samples. **B** Representative single-channel recordings of the inner mitochondrial membrane (IMM) of mitoplasts isolated from transfected HEK293 cells and corresponding open probabilities at −60 mV in a symmetric 150/150 mM KCl isotonic solution under control conditions (2 mM Ca^2+^), after adding 1 µM RTX or 10 µM CAP. **C** Data analysis of the open probability of the mitoTRPV1 channel acquired in the experiments shown in **b**, *n* = 5. Data are presented as mean ± SEM. Statistical significance of measurements compared to controls was determined at *p ≤ 0.05, **p ≤ 0.01, ***p ≤ 0.001 by one-way ANOVA. All recordings were low-pass filtered at 1 kHz. “- “ indicates the closed state of the channel. **D** Representative recordings of mitoplasts isolated from non-transfected HEK293 cells in a symmetric 150/150 mM isotonic KCl solution containing 2 mM Ca^2+^ at −60 mV. Traces are shown in control conditions (CTRL), after the application of 1 µM RTX, and in the presence of 10 µM CAP, *n* = 8. All recordings were low-pass filtered at 1 kHz. “- “ indicates the closed state of the channel. **E** Time traces of normalized Fura-2 fluorescence ratio, reflecting the cytoplasmic Ca^2+^ concentration ([Ca^2+^]_c_), in HEK293 cells expressing pmTRPV1 (light to dark green) or mitoTRPV1 (orange to red) stimulated with increasing concentrations of RTX (from 1 nM to 1 µM). **F** Inhibitory effect of capsazepine (CPZ, 10 µM) on RTX-induced [Ca^2+^]_c_ increase in HEK293 cells expressing pmTRPV1 (top) or mitoTRPV1 (down) (*n* = 3). **G** RTX dose–response curve of HEK293 cells expressing pmTRPV1 (green) or mitoTRPV1 (red) isoform and control cells (blue), inferred from the results described in **f,** and determination of the corresponding EC_50_ value using GraphPad Prism 8. **H** Western blot analysis of HEK293 cells expressing pmTRPV1-GFP (left) or mitoTRPV1-GFP (right) using anti-GFP antibody and α-tubulin antibody, as a loading control. **I** Representative time traces of normalized Fura-2 fluorescence ratio, reflecting the [Ca^2+^]_c_ in HEK293 cells expressing pmTRPV1 (green) or mitoTRPV1 (red) isoform and control cells (blue) stimulated with RTX (1 µM) in presence of extracellular Ca.^2+^, using single-cell fluorescence microscopy. Scatter plots representing the corresponding cell-by-cell maximal amplitude of RTX-response (CTRL, *n* = 101; pmTRPV1, *n* = 54; mitoTRPV1, *n* = 53)

To assess if mitoTRPV1 is functional and displays a cation channel activity, we evaluated its electrophysiological properties by performing patch-clamp recordings on mitoplasts isolated from mitoTRPV1-expressing HEK293 cells. A channel activity was detected on mitoplasts expressing mitoTRPV1 (Fig. 3B and C), but not on control mitoplasts (Fig. 3D); the latter result confirming that HEK293 cells do not express mitoTRPV1 endogenously. We assessed isolated mitochondria from sorted or unsorted transfected HEK293 cells (see patch-clamp experiments in material and methods section for more details). In the case of unsorted mitochondria, 5 out of 29 experiments showed mitoTRPV1 channel activity, whereas in sorted mitochondria, 28 out of 39 experiments exhibited channel activity (Fig. 3A). The single-channel currents were measured in a symmetric 150/150 mM KCl isotonic solution with a CaCl_2_ concentration of 2 mM. In patches, we observed a channel with a conductance of 157 ± 7 pS calculated from the mean of the current–voltage relationship (S4 Fig). No rectification of the current was observed (S4 Fig). The channel open probability (P_o_) increased with voltage, from ~ 35% at −60 mV to ~ 90% at positive voltages (S4 Fig). To confirm that the observed single-channel currents are mediated by mitoTRPV1, we used the specific agonists resiniferatoxin (RTX – 1 µM) or capsaicin (CAP – 10 µM) (Fig. 3B). A statistically significant increase in channel opening probability was observed following the application of CAP (from ~ 35% to ~ 90%) and RTX (from ~ 35% to ~ 52%) (Fig. 3C), whereas in control mitochondria from untransfected cells, the addition of RTX or CAP did not modify channel opening (Fig. 3D). Thus, these electrophysiological data demonstrated that mitoTRPV1 acts as a cation channel in mitochondria, activated by the classical TRPV1 agonists.

### mitoTRPV1 is implicated in Ca^2+^ homeostasis

To ensure that as a cation channel, mitoTRPV1 dissipates Ca^2^⁺ flows, Ca^2+^ variations were evaluated in HEK293 cells expressing the mitoTRPV1 isoform, or alternatively pmTRPV1. We used a FlexStation device to assess cellular populations or a dedicated fluorescent microscope to assess single transfected cells. Cytosolic Ca^2+^ variations ([Ca^2+^]_c_) were measured with Fura-2. The resting [Ca^2+^]_c_ was increased by pmTRPV1 expression, but not by mitoTRPV1 expression (S5B Fig). As expected, pmTRPV1 activation by RTX led to a solid dose-dependent increase in [Ca^2+^]_c_ due to a massive Ca^2+^ entry from the extracellular medium (Fig. 3F). mitoTRPV1 activation also led to a [Ca^2+^]_c_ increase, although much lower compared to pmTRPV1 activation, and both were fully inhibited by the TRPV1 antagonist capsazepine (CPZ) (Fig. 3E and F). EC_50_ values were 124.3 nM and 88.3 nM respectively for mitoTRPV1 and pmTRPV1 (Fig. 3G) and were independent of their respective expression level (Fig. 3H). These results were confirmed by a single-cell imaging method, where mitoTRPV1 activation by RTX increases cytosolic Ca^2+^ (Fig. 3I). Importantly, in a Ca^2^⁺-free buffer, activation of mitoTRPV1 leads to an increase in [Ca^2+^]_c_ suggesting that it is not related to Ca^2^⁺ influx from the extracellular environment (S5A Fig).

As the opening of the mitoTRPV1 pore therefore correlates with Ca^2+^ variations in the cell, we assessed mitochondrial matrix Ca^2+^ variations ([Ca^2+^]_mito_) using the 4mtD3cpv, a mitochondrial matrix Ca^2+^ sensor. mitoTRPV1 overexpression did not modify the resting [Ca^2+^]_mito_, while pmTRPV1 overexpression increased this parameter (S5C Fig). As expected, pmTRPV1 activation with RTX strongly increased [Ca^2+^]_mito_ related to a cytoplasmic Ca^2+^ buffering by the mitochondrial matrix (S5E Fig). Similarly, but to a much lesser extent, mitoTRPV1 activation with RTX led to a mitochondrial Ca^2+^ increase reflecting an influx of Ca^2+^ from the cytosol into the mitochondrial matrix, according to the electrochemical Ca^2+^ gradient (Fig. S5E). We then assessed Ca^2+^ variations at the mitochondrial surface hot spot ([Ca^2+^]_hot spot_) with N33D3cpv, a Ca^2+^ sensor embedded in the mitochondrial outer membrane. mitoTRPV1 expression did not modify the resting [Ca^2+^]_hot spot_, while pmTRPV1 expression increased it (Fig. S5D). The activation of pmTRPV1 with RTX further increased [Ca^2+^]_hot spot_ due to massive Ca^2+^ influx from the extracellular medium into the cytosol. Similarly, mitoTRPV1 activation by RTX increased [Ca^2+^]_hot spot_, although to a lower extent, suggesting that mitoTRPV1 indirectly promotes Ca^2+^ efflux from mitochondria (Fig. S5F). Taken together, these results suggest that mitoTRPV1 activation triggers significant but faint mitochondrial Ca^2+^ uptake and release.

### mitoTRPV1 reduces mitochondrial temperature without altering cellular respiration

**Fig. 4:**
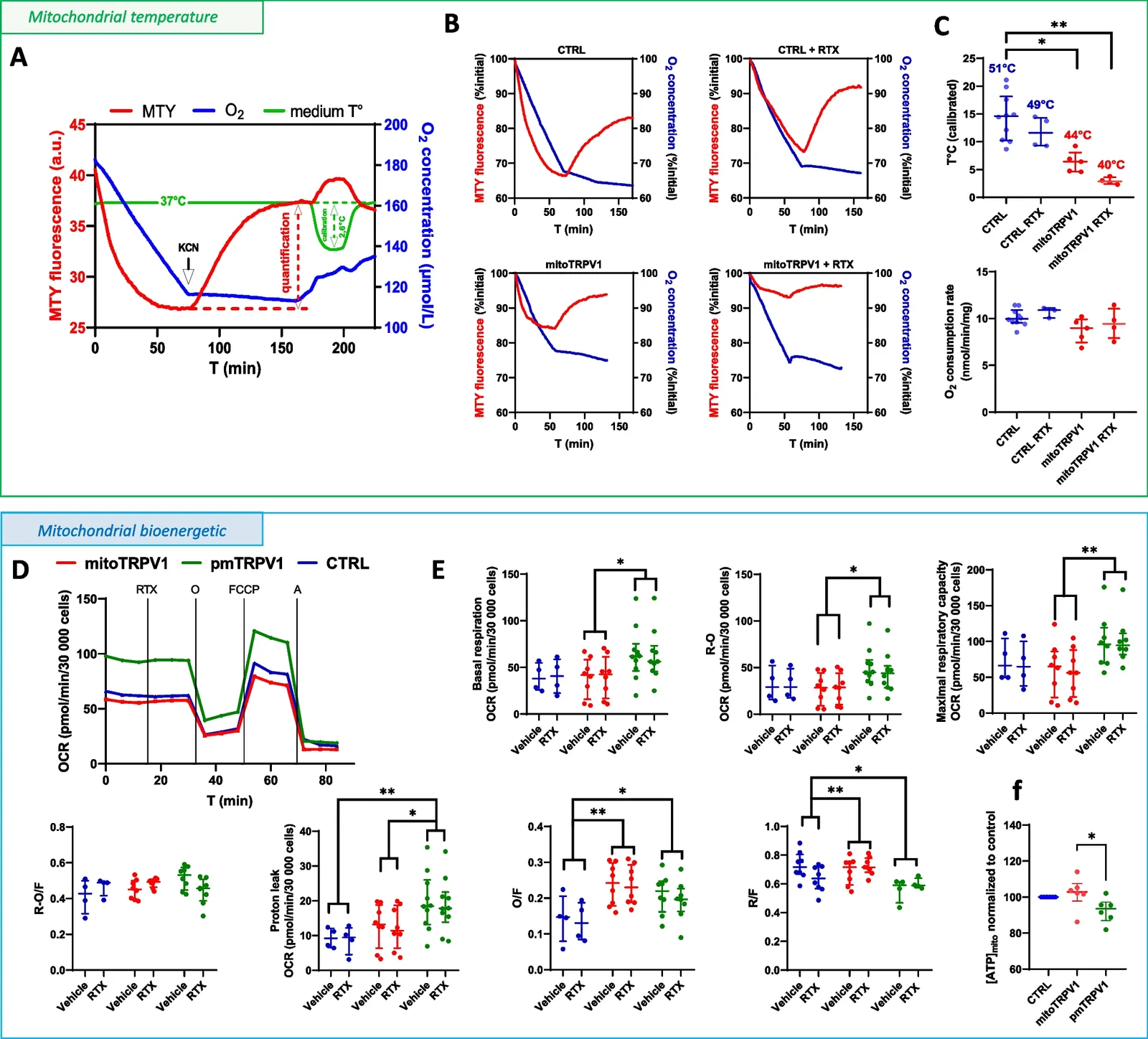
MitoTRPV1 expression and activation reduce mitochondrial temperature without affecting cell respiration and ATP synthesis. **A** Experimental design: a representative time trace of MTY fluorescence (red), O2 consumption (blue), and cell medium temperature (green) during the experience**.** Black arrow: KCN addition. **B** Representative time traces of MTY fluorescence and O_2_ consumption of CTRL HEK293 cells and cells expressing mitoTRPV1, without or with RTX (1 µM). **C** Mitochondrial temperature (ΔT°C) inferred from MTY fluorescence measurements and calibration from experiments and quantification of O_2_ consumption (nmol/min/mg) from experiments **B**. Data are from at least three independent experiments. P-value correspondences *: *p* < 0.05, **: *p* < 0.01, ***: *p* < 0.001, **** *p* < 0.0001. **D** Typical profile of O_2_ consumption rates (OCR) in intact HEK293 cells expressing pmTRPV1 (green), mitoTRPV1 (red) or control cells (blue) in a Seahorse XF96 set-up. **E** Basal respiration rates (R, b), respiration linked to ATP production (R-O, c) respiration linked to proton leak (O, d), maximal respiratory capacity (F, e), the proportion of respiration linked to proton leak compared to maximal respiratory capacity (O/F, f), the proportion of respiration linked to ATP production compared to maximal respiratory capacity (R-O/F, g), the spare respiratory capacity (R/F, h) of HEK293 cells expressing pmTRPV1 (green), mitoTRPV1 (red) or control cells (blue), in the presence of the vehicle or RTX (1 µM). **F** Mitochondrial ATP levels were measured using the mitoATeam sensor and flow cytometry. Data are from at least three independent experiments. P-value correspondences *: *p* < 0.05, **: *p* < 0.01, ***: *p* < 0.001, **** *p* < 0.0001

To evaluate if mitoTRPV1 controls mitochondrial temperature and respiration, we monitored MTY (Mito Thermo Yellow) temperature-sensitive fluorescence simultaneously with oxygen consumption in HEK293 cells expressing mitoTRPV1, activated or not by RTX (Fig. 4A-C). As previously reported for control HEK293 cells [5], the mitochondrial temperature reaches 51 °C, while the addition of RTX led to a non-significant decrease to 49 °C (Fig. 4B and C). In the same experimental conditions, we observed that mitoTRPV1 expression reduced the mitochondrial temperature to 44 °C, and further to 40 °C in the presence of RTX. Importantly, in all experimental conditions tested, the cell oxygen consumption rates were identical (Fig. 4C), witnessing that the mitochondrial temperature restriction is not associated with a decrease in mitochondrial respiration.

To confirm the absence of effect of mitoTRPV1 on oxygen consumption, we monitored mitochondrial respiration in cells expressing mitoTRPV1 or pmTRPV1 in the absence or presence of RTX, using the Seahorse XF96 device (Fig. 4D and E). Expression of pmTRPV1 increased basal respiration (R), respiration linked to ATP synthesis (R-O), maximal respiration (Fmax), respiration linked to proton leak (O), the proportion of respiration linked to proton leak compared to maximal respiratory capacity (O/F) and reduced spare respiratory capacity (R/F ratio) (Fig. 4D and E), comparatively to untransfected cells. In addition, ATP levels in mitochondria measured by flow cytometry using the ATeam sensor were also reduced in pmTRPV1-expressing cells (Fig. 4F). Conversely, expression of mitoTRPV1 did not significantly change respiration parameters (Fig. 4D and E), nor the ATP levels in mitochondria (Fig. 4F), except for the O/F ratio, suggesting a slight proton leakage. These parameters were independent of RTX activation during experiments. To confirm the H^+^ leak induced by mitoTRPV1, mitochondrial respiration was assessed in permeabilized cells expressing mitoTRPV1 using the Oroboros-O2k device (Fig. S6). This confirmed a limited increased respiration rate associated with a proton leak (EIV O), while excluding any other change of mitochondrial respiration parameter (Fig. S6). Altogether, our data suggest that mitoTRPV1 expression and further activation significantly cools down mitochondrial temperature, while maintaining the phosphorylating respirations and mitochondrial ATP levels.

### The G684V variant abolishes mitoTRPV1 functions

**Fig. 5:**
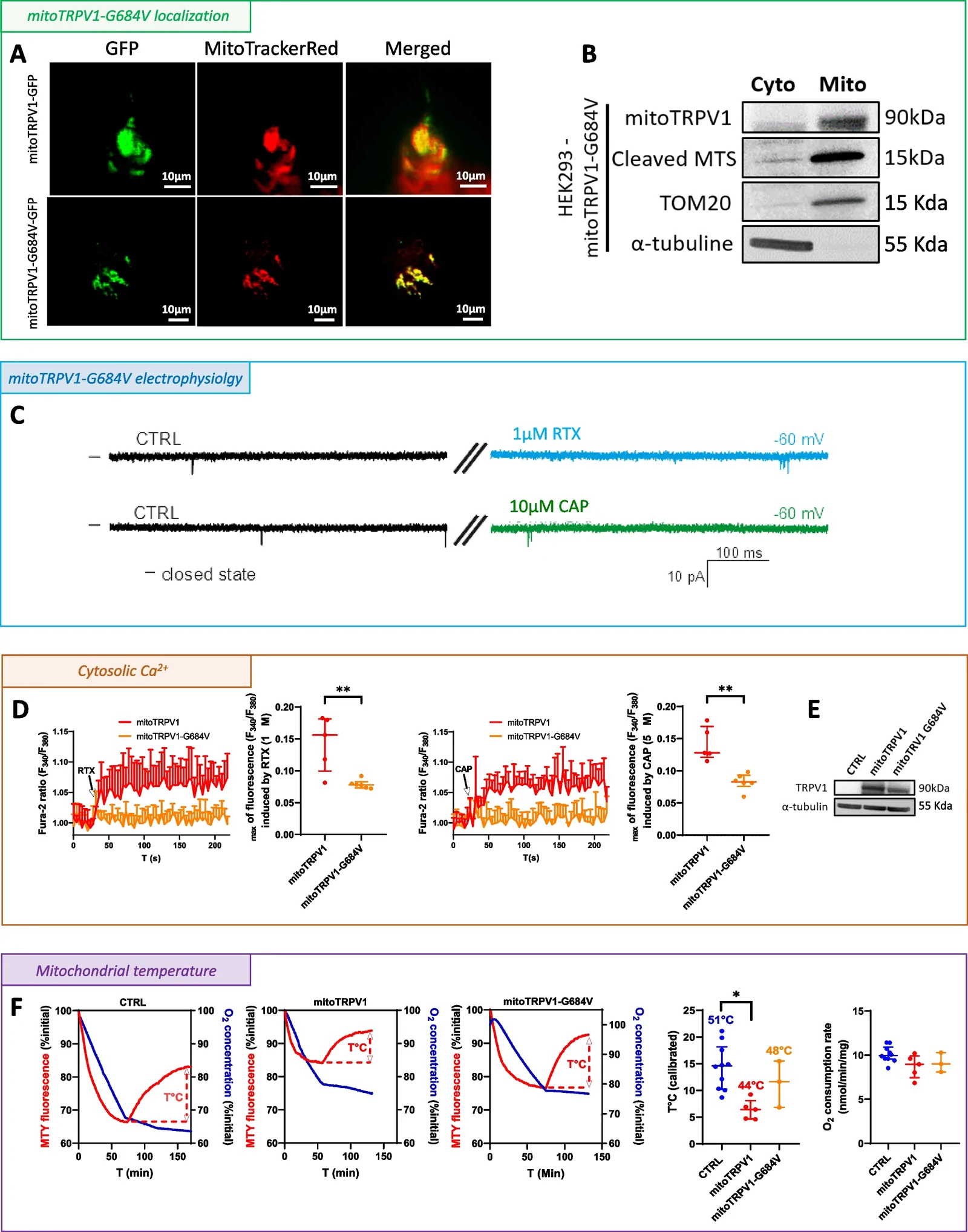
The G684V amino-acid change impairs all mitoTRPV1 functions. **A** Co-localization analysis of GFP (green) and Mitotracker (red) signals in HEK293 cells expressing mitoTRPV1-GFP or mitoTRPV1-G684V-GFP. **B** Representative Western blot analysis of cytoplasmic (left) and mitochondrial (right) fractions of HEK293 cells expressing the mitoTRPV1-G684V variant, using mitoTRPV1 antibodies targeting the mitoTRPV1 MTS. **C** Electrophysiological representation of single-channel current recorded in the mitoplasts isolated from HEK293 cells transfected with plasmid coding the mitoTRPV1-G684V mutant. Data were obtained before (CTRL), or after applying agonists (1 µM RTX or 10 µM CAP, *n* = 8). All recordings were low-pass filtered at 1 kHz. “- “ indicates the closed state of the channel. **D** Time traces of normalized Fura-2 fluorescence ratio, reflecting [Ca.^2+^]_c_, in HEK293 cells expressing mitoTRPV1 (red) or mitoTRPV1-G684V (orange) isoform stimulated with RTX (1 µM) or CAP (5 µM). Scatter plots representing the corresponding maximal amplitude of RTX-response or CAP response (mitoTRPV1, *n* = 5; mitoTRPV1-G684V, *n* = 6). P-value correspondences *: *p* < 0.05, **: *p* < 0.01, ***: *p* < 0.001, **** *p* < 0.0001. **E** Western blot of cells expressing mitoTRPV1 or mitoTRPV1-G84V, using TRPV1 antibody and α-tubulin antibody, as a loading control. **F** Representative time traces of MTY fluorescence and O_2_ consumption of HEK293 cells expressing mitoTRPV1-G684V. Mitochondrial temperature (ΔT°C) inferred from MTY fluorescence measurements and calibration from experiments and quantification of O_2_ consumption (nmol/min/mg). Data are from at least three independent experiments. P-value correspondences *: *p* < 0.05, **: *p* < 0.01, ***: *p* < 0.001, **** *p* < 0.0001

*TRPV1* pathogenic variants were associated with the development of exercise-induced hyperthermia, a severe acute pathological condition associated with body temperature overheating [17]. Consequently, we reproduced our set of experiments using a mitoTRPV1 isoform including the G684V variant identified in one patient (Fig. S1) This variant was characterized as a loss-of-function channel in the canonical form of TRPV1. First, we confirmed that the mitoTRPV1-G684 expression level in HEK293 cells is similar to that of wild-type mitoTRPV1 and is also localized to the mitochondrial network (Fig. 5A and B). Then, we evaluated the electrophysiological properties of this mutated isoform and evidenced first that mitoplasts isolated from mitoTRPV1-G684V-expressing cells do not exhibit any channel activity and second that TRPV1 agonists do not induce any channel opening (Fig. 5C), in marked contrast with mitoplast including wild-type mitoTRPV1 (Fig. 3D). In addition, we found that the expression of mitoTRPV1-G684V (Fig. 5E) displays no significant activity on mitochondrial temperature (Fig. 5F), and that it is completely ineffective in promoting cytosolic Ca^2+^ increase after stimulation by CAP or RTX (Fig. 5D). Thus, our results confirm that the G684V variant is a loss-of-function variant that abolishes mitoTRPV1 opening and consequently its channel activity, and that the cooling activity of mitoTRPV1 is intimately coupled to the Ca^2+^ homeostasis through a mechanism that must be further explored.

## Discussion

Understanding mitochondrial thermoregulation is a crucial challenge for promoting the maintenance of mitochondrial physiology, as well as the surrounding cytoplasmic and nuclear compartments, and more broadly, for the entire organism. Recent studies have reported very high mitochondrial matricial temperatures not just as a by-product of metabolite oxidation, but as a major function designed to support normal cell function [5, 6]. We reasoned that excess temperature could jeopardize cell physiology and should be downregulated by a cooling system to prevent dramatic overheating [29]. In this respect, we screened *TRP* open reading frames coding for isoforms potentially localized to mitochondria that could act as a thermostat.

We identified mitoTRPV1, a novel *TRPV1* mitochondrial isoform including an effective N-terminal targeting sequence driving TRPV1 to the inner mitochondrial membrane. MitoTRPV1 is encoded by a yet uncharacterized TRPV1 mRNA consisting in a + 1 overlapping ORF starting in exon1 and spanning exon2, which is then reconnected to the consensual TRPV1 ORF by the maintenance of the small 70 nt-long coding intron2. This molecular process leading to two overlapping ORFs is exceptional in the nuclear genome of higher eukaryotes, whereas it is much more common in viral genomes and plastids [30, 31]. The mitoTRPV1 mRNA variant, like most genes encoding mitochondrial proteins, is ubiquitously expressed in all human tissues, although with a mean 26-fold lower abundance compared to the conventional pmTRPV1 mRNA, a fact that we also evidenced in human myoblasts and muscle biopsy, but not yet in other tissues. This mitoTRPV1 mRNA encodes a 150 aa amino-terminal MTS that is cleaved after mitochondrial import, which we demonstrated to be both mandatory and sufficient for embedding TRPV1 core domains into the inner mitochondrial membrane, as for most mitochondrial ion channels [32]. Future studies aiming to identify the orientation of mitoTRPV1 in the IMM will be important for evaluating how ATP concentrations and mitochondrial pH, two strong modulators of TRPV1 [33, 34], can monitor its channel activity and temperature sensitivity. Moreover, the N-terminal modification of mitoTRPV1 results in the deletion of the first ankyrin repeat domain (Ank) and may affect mitoTRPV1 regulation as these domains interact with intracellular modulators such as ATP and calmodulin [35]. Thus, the missing 150 amino acids in the N-terminal domain of mitoTRPV1 should not impair the tetramerisation of the channel, which depends on the C-terminal domain [36]. Importantly, we found that the TRPV1 MTS is conserved only among mammal species, but not in other vertebrate phyla, even among birds that share homeothermy with mammals, supporting the concept that divergent molecular processes led separately to homeothermy in these two phyla [37, 38]. In addition, we identified three mammalian species that cannot encode TRPV1 MTS, including the platypus, which exhibits unorthodox thermal regulation with a low body temperature (32 °C) and intolerance to temperatures higher than 25 °C [39], and the naked mole rat, which lives underground at a constant temperature.

Further proof of mitoTRPV1 localisation and activity in the IMM was obtained by patch-clamp analyses on mitoplasts purified from HEK cells expressing mitoTRPV1, which were not detected in mitochondria from untransfected control cells, indicating that the IMM does not contain other channels endogenously, as BKCa. Measured currents were induced by the TRPV1 agonists RTX and CAP, as expected. They showed a conductance of 157 pS, without voltage-dependency at the single-channel level, in agreement with data obtained for the conventional pmTRPV1 isoform [40]. This contrasts with the measurement of other mitochondrial ion channels activity, such as mitoK_ATP_ that exhibited a conductance twice higher than the pmK_ATP_ channel (Bednarczyk et al., 2008); taking into account that the channel conductance depends primarily on the ionic strength of the experimental solutions and could further be modulated to the presence of partner proteins and the lipid composition specific of the IMM.

We then demonstrated that the expression of mitoTRPV1, in phosphorylating condition, restrains the increase of the mitochondrial temperature to 44 °C, a temperature compatible with the conventional TRPV1 isoform activation [10]. In contrast, untransfected control HEK cells, which do not express TRPV1 isoforms, showed a mitochondrial temperature of 51 °C, as previously reported [5, 6]. Further preincubation with RTX reduced the maximal mitochondrial temperature to 40 °C without any inhibition of respiration rates in cells expressing mitoTRPV1, while remaining ineffective in control cells. These data provide strong evidence that mitoTRPV1 function and activation consist in preventing mitochondrial overheating. Surprisingly, the cooling effects associated with mitoTRPV1 had no simultaneous effect on mitochondrial respiration parameters except for a slight increase in H^+^ leak. The mitochondrial contribution to heat production is usually primarily through OXPHOS reactions [41], which makes it paradoxical to observe a mitochondrial cooling process without change in respiration efficacy. This prompts us to propose that mitoTRPV1 promotes heat dissipation or thermolysis rather than a restriction of the thermogenesis by the inhibition of the Krebs cycle and OXPHOS activities.

Although the proper mechanism responsible for thermolysis remains to be fully demonstrated, we postulate that the circulation of Ca^2+^ fluxes induced by mitoTRPV1 activation contributes to this process by facilitating Ca^2+^ uptake and release from mitochondria, and consequently, exchanging heat from mitochondria to their environment. To support this hypothesis, we demonstrated that at a cellular level, mitoTRPV1 acts as a Ca^2+^ channel. It holds basic pharmacological features similar to the conventional pmTRPV1 isoform: comparable EC_50,_ RTX sensitivity and CPZ antagonism [10], although both TRPV1 isoforms (mito and pm) are embedded in membranes with highly different lipid and protein compositions [42]. Thus, our results question the proper mechanism responsible for mitochondrial thermolysis triggered by mitoTRPV1 activation. The role of mitoTRPV1 is clearly related to intracellular Ca^2^⁺ homeostasis in our experimental conditions: it contributes – directly or indirectly – to a faint increase in Ca^2^⁺ in three cellular compartments, namely the cytosol, MAMS and mitochondrial matrix. Recently, it was shown that the activation of a mitochondrial TRPV1 in endothelial cells leads to a Ca^2+^ mobilization from ER stores to the mitochondrial matrix, independently of external Ca^2+^ [28]. Our data suggests a comparable mechanism, promoting mitoTRPV1 as a Ca^2+^ cycling regulator, whereby Ca^2+^ would circulate between internal compartments, prompting extrusion of heat from mitochondria [43], promoting mitoTRPV1 as a pharmacological target for thermoregulatory mechanisms These finding can be linked to recent theoretical studies that highlighted the potential contribution of ion transport to local thermal phenomena within biological membranes. Using an electrochemical model, Wazawa and Nagai showed that transmembrane ion fluxes can be associated with either heat production or heat absorption depending on the direction of transport relative to the electrochemical gradient, and suggested that such processes may contribute to intracellular thermal regulation, including mitochondrial thermogenesis [44]. These observations further support the idea that membrane proteins involved in ion transport participate in the establishment and modulation of local temperature microenvironments within cells. This hypothesis remains speculative and deserves investigations at the molecular and thermodynamic levels.

The function of mitoTRPV1 as a thermal sensor is further consistent with clinical and genetic reports that evidenced pathological variants in the *RYR1*, *SERCA* and *TRPV1* genes predisposing to life-threatening hyperthermia [16, 17, 45, 46]. Each pathological variant affecting these genes would impair intracellular Ca^2+^ fluxes, in particular in-between the mitochondria and the ER, and consequently compromise mitochondrial cooling, ultimately leading to mitochondrial, cellular, organ and body overheating. This process is further reinforced by results obtained with the mitoTRPV1 isoform including the missense G684V mutation, which abolishes the channel opening and Ca^2+^ transfer, leading to a predisposition to exertional heat stroke in humans [17]. Indeed, our experimental approach evidenced that the mitoTRPV1-G684V isoform is ineffective in generating Ca^2+^ fluxes and in cooling mitochondria, thus establishing a direct and strong correlation between mitoTRPV1 activity, Ca^2+^ homeostasis and mitochondrial heat dissipation.

Nevertheless, this work presents some limitations. First, we have not been able to demonstrate the physiological presence of mitoTRPV1 in mitochondria, due to the absence of relevant antibodies recognizing the N-terminal region of mitoTRPV1. This prompted us to transiently express mitoTRPV1 in HEK cells that do not endogenously express TRPV1. As a result, our findings might not reflect the basic function and regulation of mitoTRPV1. In this respect, further studies in physiological models are required to demonstrate the in vivo relevance of mitoTRPV1 function. Another limitation lies in the use of the MTY dye to estimate mitochondrial temperature. The reliability of this dye remains a strong matter of debate, further supported by the fact that the measurement of mitochondrial temperature is an emerging field, which suffers from the absence of any method reaching a clear consensus. Despite these limitations, the overall consistency of the multiple independent observations supports the biological relevance of our findings. In particular, the evolutionary conservation of mitoTRPV1 among homeothermic mammals, the validation of the observed TRPV1-dependent effects using a mitoTRPV1 mutant associated with hyperthermia, the electrophysiological properties of mitoTRPV1 and the detection of its transcript in human skeletal muscle, together providing convergent evidence supporting the role for mitoTRPV1 in the regulation of mitochondrial temperature.

Altogether, we propose that in mammals, the mitoTRPV1 isoform triggers a process responsible for mitochondrial heat dissipation, when the mitochondrial temperature reaches 43–44 °C, by promoting a Ca^2+^-dependent cooling circulation between mitochondria and their direct environment, possibly involving the endoplasmic/sarcoplasmic reticulum. In the future, it will be necessary to test the mitochondrial thermostat activity in physiological and pathological conditions, notably in muscle fibers and neurons, which are the principal targets of overheating. Finally, the characterization of mitoTRPV1 isoform opens novel avenues for identifying pharmacological agonists and antagonists specific to each TRPV1 isoform, thereby enabling an improved and separated management of nociception or body temperature in humans.

To conclude, we have identified a human TRPV1 variant localized in mitochondria due to the presence of a mammalian-specific mitochondrial targeting sequence, which accounts for an RTX-sensitive Ca^2+^ transport through the inner mitochondrial membrane. The ectopic expression of this mitoTRPV1 protein recapitulates the electrophysiological properties and pharmacological profile of pmTRPV1 and represents a novel mechanism contributing to mitochondrial thermoregulation and homeothermy.

## Materials and methods

### Antibodies and reagents

The following antibodies were used: anti-GFP (BD Biosciences Cat# JL-8, RRID:AB_2314359), anti-6X-His (Thermo Fisher Scientific Cat# MA1-21315, RRID:AB_557403), anti-α-tubuline (Abcam Cat# ab52866, RRID:AB_869989), anti-TOMM20 (Abcam Cat# ab78547, RRID:AB_2043078), anti-TIMM23 (Abcam Cat# ab116329, RRID:AB_10903878), anti-SERCA (Abcam Cat# ab2817, RRID:AB_2061427), anti-TFAM (Abcam Cat# ab155240), anti-VDAC (Abcam Cat# ab14734, RRID:AB_443084), anti-GRP75 (Abcam Cat# ab2799, RRID:AB_303311), ECL anti-mouse HRP-linked (ThermoFisher Cat# 45–001–275), ECL anti-mouse HRP-linked (ThermoFisher Cat# 45–001–275). Additionally, two homemade TRPV1 antibodies targeting the C-terminal part of TRPV1 (epitope: GSLKPEDAEVFKSPAASGEK) or the MTS part of mitoTRPV1 (epitope: ARPSQSALLSPSRGQET) were generated by Genosphere Biotechnology.

The following reagents were used: NucleoSpin RNA set for NucleoZOL kit (Macherey–Nagel Cat#740406.50), iScript Reverse Transcription Supermix (Biorad Cat#1708841), Phusion high-fidelity master mix with GC buffer (ThermoFisher Cat#F532L), PCR clean up (Macherey–Nagel Cat#740609), Infusion snap assembly (Takara Bio Cat#638943), CloneAmp™ HiFi PCR (Takara Bio Cat#639298), BigDye™ Terminator v3.1 (ThermoFisher Cat#4337455), Ham’s F10 (Life Technologies Cat#11550043), Ultroser G (Pall-sartorious Cat#15950–017), Penicillin–streptomycin (Life Technologies Cat#15140122), DMEM low glucose (Life Technologies Cat#31885023), TrypLE (Life Technologies Cat#12605–010), DMEM (Pan Biotech Cat# P04-03500), L-glutamine (ThermoFisher Cat#25030081), DPBS (PAN Biotech Cat# P04-36500), Trypsin (PAN Biotech Cat# P10-023100), Hank’s Balanced Salt Solution (ThermoFisher Cat#14025092), Avalanche®-Omni (EZ Biosystems Cat#EZT-OMNI-1), DharmaFECT (Horizon Discovery Cat#T-2001–03), Mitotracker Red CMXRos (ThermoFisher Cat# M46752), Fura-2 AM (ThermoFisher Cat#F1221), Pluronic^®^-F127 acid (ThermoFisher Cat#P3000MP), pcDNA –4mtD3cpv (RRID:Addgene_36324), Proteinase K (ThermoFisher Cat#EO0491), Digitonin (ThermoFisher Cat#407560050), Triton X-100 (Sigma Aldrich Cat#X100-100ML), PMSF (ThermoFisher Cat#36978), Trichloroacetic acid (Sigma Aldrich Cat# T6399), Capsaicin (Alomone labs Cat#C-125), Resiniferatoxin (Alomone labs Cat#R-400), Diméthylsulfoxyde (Sigma Aldrich Cat#D8418-50ML), Oligomycin (Sigma Aldrich Cat#75351-5MG), Carbonyl cyanide 4-(trifluoromethoxy)phenylhydrazone (Sigma Aldrich Cat#C2920-10MG), Antimycin-A (Sigma Aldrich Cat#A8674-25MG), Potassium cyanide (Sigma Aldrich Cat#207810-25G), Sodium pyruvate (Sigma Aldrich Cat# P5280-25G), Malic acid (Sigma Aldrich Cat#02288-10G), Adénosine 5′-diphosphate sodium salt (Sigma Aldrich Cat#A2754-100MG), Succinic acid (Sigma Aldrich Cat#797987-100G), Rotenone (Sigma Aldrich Cat#R8875-1G), Sodium Ascorbate (Sigma Aldrich Cat#1613509-200MG), N,N,N'N'-tetramethyl-1,4-phenylenediamine (Sigma Aldrich Cat#T3134-5G), Sodium azide (Sigma Aldrich Cat#S2002-5G).

### Human myoblast culture and analysis

Investigations on patient material were performed after the signature of an informed consent according to the French regulation and have received approval from the local ethical committee (Comité de Protection des Personnes-Sud-Est, France). Immortalized human satellite cells (myoblasts) have been produced as described previously [47] from a 25-year-old control individual [48]. Immortalized human satellite cells were amplified in a proliferation medium composed of F-10 nutrient medium supplemented with 20% fetal bovine serum (FBS), 2% Ultroser G, and 2% Penicillin–streptomycin. Myoblasts are kept at a confluence 50%–60% max. Differentiation into myotubes was induced by a shift to differentiated medium DMEM low glucose supplemented with 2% heat-inactivated horse serum and 1% penicillin–streptomycin. The cells were collected with TrypLE at proliferation and 3 and 6 days of differentiation and RNA extracted with the NucleoSpin RNA set for NucleoZOL kit following the manufacturer’s recommendations for a large number of cells (2,000,000 cells). RNA has been retro-transcribed to cDNA using the iScript Reverse Transcription Supermix for RT-qPCR according to the manufacturer’s recommendations. PCR amplification of the cDNA was performed with Phusion high-fidelity master mix with GC buffer. The different primer pairs are the following, for pmTRPV1 Fw: *AAGTTCACCATCGGCATGGG*; Rv: *AAGGAAGCTCTTCTCCGTGTC*. For mitoTRPV1, Fw: *TGGAGACCCTAACTCCAGGC*, Rv: *CAGGGTCTGAAAGACATAAGGGA*. All reactions were run on a thermocycler with the following amplification step: For pmTRPV1: 98 °C – 5 min; 35 cycles composed of 98 °C – 30 s, 65 °C – 15 s, 72 °C – 20 s; 72 °C – 2 min. For mitoTRPV1: 98 °C – 5 min; 30 cycles composed of 98 °C – 30 s, 57 °C – 15 s, 72 °C – 20 s; 72 °C – 2 min. The PCR products were then separated on a 2% agarose gel. DNA bands of interest were excised and purified with the gel and PCR clean up kit with a final elution volume of 20 mL and submitted to Sanger sequencing (Eurofins Genomics).

### Human muscle biopsy sequencing

Samples were obtained from soldiers of the French national cohort recruited between January 2004 and December 2006 by the Military Hospital Lavéran reference center for EHS. Patients were included based on a characterized EHS crisis [17]. Genetic analyses were performed after written informed consents were obtained. RNA was extracted from a skeletal muscle biopsy and reverse-transcribed according to the manufacturer's protocol (Transcriptor, Roche Applied Science, USA). Sanger sequencing of the 5’ region was performed with the primer: TRPV1_F1 CCG GTC AGT CAC TTA GTC GT.

### DNA analysis and MTS prediction

All TRPV1 protein and nucleotide sequences were collected from the NCBI database (https://www.ncbi.nlm.nih.gov/, visited on 02/06/2025) and analyzed with the MitoProt-II tool [49] (https://ihg.helmholtz-munich.de/ihg/mitoprot.html, Last Update December 2006, version 1.101) to predict the presence of an N-terminal mitochondrial targeting sequence (MTS) and its cleavage site. For the MTS prediction in animals for which no TRPV1 mRNA variant is described in protein databases, *TRPV1* gene sequences were collected from the NCBI DNA database and analyzed by a home-made algorithm screening all the open reading frames (ORF) for the presence of an MTS sequence in the first coding exons. The highest score was retained for each species and is shown in Fig. 1F.

Using the TRPV1 protein sequences collected from the NCBI database and a home-made algorithm, a multiple alignment was performed using ClustalOmega (v1.2.3) [50]. IQ-tree (version 2.0.3) [51] was used to generate the complete phylogenetic tree. ModelFinder [52], included in IQ-tree, was used to assess the best-fit substitution model. A maximum likelihood (ML) tree was generated by IQ-tree using the HIVw + F + G4 substitution model. Bootstrapping was performed by 1,000 iterations for Ultra-Fast Bootstrapping UFBoot. The consensus tree was visualized using the interactive tree of life v5 (iToL, https://itol.embl.de) [53].

### Cloning

All primers are described in S1 Table. Plasmid encoding pmTRPV1 (pcDNA5/FRT-TRPV1) was a gift from Aubin Penna (4CS labs—CNRS UMR 6041, Poitiers, France) and was used to clone mitoTRPV1 (pcDNA5/FRT-mitoTRPV1) using the Infusion Cloning method. First, pcDNA5/FRT-TRPV1 was linearized by inverse PCR using A-for and B-for primers. Then, two synthetic complementary oligonucleotides corresponding to *TRPV1* intron2 were obtained from ThermoFischer and used to reconstruct the double-strand insert: B-for and B-rev. The insert was cloned using a vector/insert ratio 1/10 to generate an intermediate plasmid with an ORF coding for mitoTRPV1. Then, this plasmid was linearized by inverse PCR using the primers C-for and C-rev to delete the sequence located upstream of the mitoTRPV1 alternative start codon. The plasmid was re-circularized to obtain the final plasmid coding for mitoTRPV1 (pcDNA5/FRT-mitoTRPV1). The same method was used to construct the plasmid coding for mitoTRPV1-G684V mutant, using G684V-for and G684V-rev primers and the GFP, mCherry or 6X-His tagged versions of TRPV1, mitoTRPV1 or mitoTRPV1-G684V using D-for/D-rev, GFP-for/GFP-rev, mCherry-for/mCherry-rev and 6XHis-for/6XHis-rev primers. All plasmids were validated by Sanger sequencing.

### Cell culture and transfection

HEK293 and MCF7 cells were cultured in Dulbecco’s modified Eagle medium with 10% fetal bovine serum (FBS), 4.5 g/L glucose and 1 mM L-Glutamine at 37 °C and 5% CO_2_ and were routinely tested for Mycoplasma infections. The day before transfection, cells were seeded on different supports to obtain 70%−80% confluence 24 h later. Cell transfections were performed as previously described for individual cell Ca^2+^ imaging and ATP measurements [54]. Transfections were performed using Avalanche®-Omni or DharmaFECT. Cells were transfected with a previously incubated transfection mix that contained 500 µL of serum-free DMEM, 2 µL of Dharmafect Duo or Avalanche®-Omni, and 2 µg of DNA plasmid (a mix of 1 µg/1 µg for co-transfection). Media were changed after 24 h and cells were analyzed 48 h after the transfection.

### Patch-clamp experiments

Patch-clamp experiments on mitoplasts were performed following previously described protocols [55, 56]. Briefly, mitoplasts were prepared from mitochondria isolated from wild-type HEK293 cells (not transfected), HEK293 cells expressing mitoTRPV1-GFP or mitoTRPV1-G684V-GFP mutant.

Before mitochondria isolation, cells were sorted by flow cytometry. 48 h post-transfection, cells were gently detached using trypsin, washed once with PBS, and resuspended in PBS supplemented with 1% FBS. Cells were then analyzed and sorted on a FACSAria II flow cytometer (BD Biosciences, San Jose, CA) based on GFP fluorescence. Transfection efficiency prior to sorting ranged from 10 to 20%. Post-sort analysis confirmed that 95% of the sorted cells were GFP-positive, i.e. expressing mitoTRPV1. This sorting protocol was used to minimize the percentage of patches without detectable channel activity ("empty patches"). This step enabled to move from a percentage of 17.2% of mitoTRPV1 channel currents (possessing a conductance of ∼157 pS) relative to the total number of patch-clamp experiments performed to 71.8% (Fig. 3A). Mitochondria were placed in a hypotonic solution (10 mM MOPS, 2 mM CaCl_2_, pH = 7.0) for approximately 2–4 min to induce swelling and rupture of the outer mitochondrial membrane. Subsequently, a hypertonic solution (750 mM KCl, 10 mM MOPS, 2 mM CaCl_2_, pH = 7.0) was added to restore the isotonicity of the medium. The patch-clamp pipette was filled with an isotonic solution containing 150 mM KCl and 10 mM MOPS, 2 mM CaCl_2_ (pH = 7.0). As previously described [57], this symmetrical condition is adapted for patch-clamp mitochondria from HEK cells. For pmTRPV1 channel, selectivity is described in direct divalent cations, with a focus on Ca^2+^_ext_ (Caterina et al., 1997). We decided to increase the [Ca^2+^]_ext_ to 2 mM for patching mitochondria in mitoTRPV1-expressing HEK cells. This Ca^2+^-containing external solution was used just before patch-clamp measurement. After osmosis and patching, the IMM is already torn. Using inside-out configuration of patch-clamp, we measure ion transport through the channel independently of mitochondrial physiology.

Mitoplasts were identified by their size, round shape, transparency, and distinct "cap," which set them apart from the cellular debris in the preparation. CAP and RTX were added as dilutions in an isotonic solution containing 2 mM CaCl_2_. A perfusion system containing a holder with a glass tube, a peristaltic pump, and Teflon tubing was used to apply these substances. The mitoplasts at the tip of the measuring pipette were transferred into the openings of a multibarrel “sewer pipe” system in which their outer faces were rinsed with the test solutions.

The electrical connection was made using Ag/AgCl electrodes and an agar salt bridge (3 M KCl) as the ground electrode. Membrane potential is reported relative to the mitochondrial matrix side. The current was recorded using a patch-clamp amplifier (Axopatch 200B, Molecular Devices Corporation, USA). The pipettes, made of borosilicate glass, had a 10–20 GOhm resistance and were pulled using a PC-100 Narishige puller.

The currents were low-pass filtered at 1 kHz and sampled at 100 kHz. The traces of the experiments were recorded in inside-out single-channel mode (mitoplast patch-clamp). The recordings shown are representative of the most frequently observed conductance under each experimental condition. Channel conductance was calculated from the current–voltage relationship (−60 to + 60 mV). In mitochondrial patches, the physiological membrane potential of mitochondria (−180 mV) is unattainable, as the membrane ruptures very quickly at such a high external potential. The probability of channel opening was determined using the single-channel search mode of the Clampfit software. Data from the experiments are reported as the mean values ± SEM. In figures showing single-channel recordings, “-” indicates the closed state of the channel.

### Fluorescent microscopy live imaging

Cells were seeded and transfected in a 4-well µ-slide 4 (Ibidi). For live imaging, cells were loaded with 50 nM Mitotracker Red CMXRos for 15 min at 37 °C. After rinsing with fresh medium, µ-slide were placed under a microscope NIKON ECLIPSE Ti-E (Nikon Instruments Europe) equipped with a camera Andor NEO sCOMS. Image acquisition and analysis were performed with Metamorph 7.7 software (Molecular device). 30 image planes were acquired along the Z-axis at 0.1 μm increments, and images were iteratively deconvolved using Huygens Essential® software (Scientific Volume Imaging, Hilversum, The Netherlands).

### Mitochondria isolation

All cell fractionation experiments were performed according to a method already described [58]. Briefly, mitochondria were isolated by suspending cells in an ice-cold isolation buffer (Mannitol 225 mM, Sucrose 75 mM, HEPES 10 mM, EDTA 10 mM, DTT1 mM). Cells were broken with a glass/Teflon Potter homogenizer by 100 movements. Cell suspensions were centrifuged at 800 g for 5 min at 4 °C. The supernatant was centrifuged at 6000 g for 10 min at 4 °C. The mitochondria pellets were re-suspended in an isolation buffer and used for proteinase K protection assay, Western blot, or subsequent centrifugations for Mitochondrial Associated Membranes (MAMs) isolation.

### Proteinase K protection assay

Mitochondria from HEK293 cells expressing mitoTRPV-6X-His were isolated and treated with increasing concentrations of digitonin (0%–0.15%) and a constant concentration of Proteinase-K at 50 µg/ml for 15 min at room temperature. Samples without proteinase-K or with 1% Triton X-100 served as controls. Proteinase-K was inactivated by adding 100 µM PMSF, followed by incubation with ice-cold 10% trichloroacetic acid (TCA) on ice for 15 min. After centrifugation (10.000 g, 15 min, 4 °C), TCA precipitates were dissolved in an SDS-page loading buffer, and samples were analyzed by Western blot.

### Intracellular Ca^2+^ measurements

Cytosolic Ca^2+^ imaging experiments were carried out on populations of cells with a FlexStation® 3 Benchtop Multi-Mode Microplate Reader. HEK293 expressing pmTRPV1 or mitoTRPV1 were plated at a density of 50,000 cells by well (96 wells black/transparent bottom plate) in culture medium. Twenty-four hours after plating, cells were incubated for 60 min at RT with 4 µM Fura-2 AM and Pluronic®-F127 acid (0.02%) in freshly prepared buffer composed of Hank’s Balanced Salt Solution (HBSS) supplemented (in mM): 2.5 CaCl_2_, 1 MgCl_2_ and 10 HEPES-K (pH 7.4). After washing, cells were incubated in the buffer for 60 min for complete de-esterification of the dye. Plates were illuminated at 340 and 380 nm excitation wavelengths, and the fluorescence emission spectra were recorded at 510 nm. After a 30 s baseline, TRPV1 activators, including RTX, were automatically injected, and the fluorescence emission spectra were monitored for 320 s at an acquisition frequency of 0.25 Hz. All experiments were performed in triplicate at least twice. Data was analyzed using the SoftMax Pro 5.4.1 software (Molecular Devices, Sunnyvale, CA, USA).

For individual cell Ca^2+^ imaging, HEK293 cells were plated on glass coverslips (24 mm diameter) in a 6-well plate. For cytosolic Ca^2+^ measurements, cells transfected with mCherry-tagged plasmid (expressing pmTRPV1-mCherry or mitoTRPV1-mCherry) were incubated for 30 min at RT with 2 µM Fura-2 AM in calcium-containing buffer (CCB) or in calcium-free buffer (CFB). CCB consists of (mM) 140 NaCl, 5 KCl, 1 MgCl2, 10 HEPES, 10 glucose, and 2 CaCl2, adjusted to pH 7.4 and CFB consists in CaCl2 replacement by 1 mM EGTA. For all other Ca^2+^ measurements, HEK293 cells were co-transfected as described in the section “Cell culture and transfection” section. 4mtD3cpv or N33D3cpv were used to measure mitochondrial matrix and mitochondrial surface hot spot Ca^2+^ concentrations, respectively. cDNA-4mtD3cpv was a gift from Amy Palmer and Roger Tsien [59]. N33D3cpv was a gift from Yves Gouriou [60]. Experiments were performed at room temperature (RT) in CCB. Glass coverslips were mounted on a magnetic chamber (Chamlide) and placed on a DMI6000 inverted wide-field microscope (Leica Microsystems, Wetzlar, Germany). Images were acquired with an Orca-Flash 4.0 Scientific CMOS camera (Hamamatsu, Photonics, Shizuoka, Japan) using a 40X oil-immersion objective and a Lambda DG-4 + filter (Sutter Instruments, Novato, CA, USA). mCherry fluorescence was excited at 572/35 nm, Fura-2 AM at 340 and 380 nm, and their respective fluorescent emissions were measured at wavelength 610 nm and 510 nm, respectively. 4mtD3CPV and n33D1CPV were excited at a wavelength of 430 nm, and their emissions were collected at 480 nm and 530 nm. Images (1024 × 1024 pixels) were taken with 5 s (Fura-2 AM and erGAP1) or 2 s time intervals (N33D3cpv and 4mtD3cpv). Fluorescence ratios of mCherry-positive cells were analyzed with MetaFluor 6.3 (Universal Imaging) after removing background fluorescence.

All Ca^2+^ kinetics display fluorescence intensities as follows: Fx = F-F0 for single cell experiments, or Fx = F/F0 for cellular populations, where F corresponds to the measured fluorescence and F0 to the baseline fluorescence. Variations of Ca^2+^ concentrations are shown in response to RTX treatment as either area under the curve (AUC) or fluorescence peak (ΔF).

### Mitochondrial respiration with Seahorse^®^ XF96

Mitochondrial respiration of transfected cells was performed using the Seahorse XF96 device (Agilent), as described by [61]. Briefly, HEK cells transfected for 48 h were plated in a 96-well Seahorse plate at 30,000 cells per well in a culture medium. After 5 h, the culture medium was replaced by a non-buffered assay medium (pH = 7.4) containing 4.5 g/L glucose and 1 mM L-Glutamine, and cells were incubated at 37 °C (0% CO_2_) for 1 h. Cell oxygen consumption was measured during drug injections. Injection #1 was for RTX or DMSO, injection #2 for oligomycin (2 µg/ml), injection #3 for FCCP (50 to 1000 nM), and injection #4 for antimycin-A (2 µg/ml). Data analyses were performed using the Wave software (Agilent). Cell numbering was used for data normalization.

### Mitochondrial respiration with Oroboros^®^ O2k

Mitochondrial oxygen consumption measurements were performed at 37 °C and atmospheric pressure using a high-resolution oxygraph (O2k, Oroboros^®^ Instrument, Innsbruck, Austria). Respiration rates on permeabilized cells were measured in respiratory buffer RB (10 mM KH_2_PO_4_, 300 mM mannitol, 10 mM KCl, 5 mM MgCl_2_, 0.5 mM EGTA, and 1 mg/ml serum albumin bovine, pH 7.4) using substrates of Complex I (CI) complexe I and II (CI + CII) and complex II (CII) as follows. First, state 2 (non-phosphorylating) respiration was measured after adding 2.5 mM pyruvate and 5 mM malate. Second, the CI-linked maximal phosphorylating respiration was stimulated by saturating ADP concentration (1.5 mM). Succinate (10 mM) was then added to measure the combined CI and CII-linked respiration. Rotenone (5 μM) was used to inhibit CI activity and obtain maximal CII-linked respiration. Third, oligomycin (F0F1-ATP synthase inhibitor, 4 μg/ml) and FCCP (carbonyl cyanide *p*-trifluoromethoxyphenylhydrazone, a mitochondrial uncoupler, 1 μM) were sequentially added to ensure that cells were fully permeabilized. Finally, antimycin-A (2 μg/ml) was added to inbit complex III, before the addition of 4 mM ascorbate (Asc) and 0.3 mM N,N,N'N'-tetramethyl-1,4-phenylenediamine (TMPD), an artificial electron donor for cytochrome c oxidase. Finally, this one was inhibited with 1 mM potassium cyanide (KCN) and 2 mM sodium azide (Azide) to determine unspecific oxidations.

### Mitochondrial ATP measurement by flow cytometry

All experiments were performed on a Fortessa X‐20 cells analyzer instrument (BD Biosciences) equipped with 4 lasers: violet laser 405 nm, blue laser 488 nm, Yellow-green laser 561 nm, and red laser 640 nm. The vector expressing mitoATeam, the mitochondrial ATP-sensitive FRET probe, was kindly supplied by Hiroyuki Noji [62]. Ratiometric analysis of mitoATeam was measured by excitation at 405 nm and 488 nm and emission at 525/50 nm and 530/30 nm for CFP and YFP, respectively. mCherry fluorescence was measured by excitation at 561 nm and emission at 610/20 nm. The gating of the HEK cells was based on two combined parameters. First, events were gated for singlets based on FSC-height (FSC-H) and FSC-area (FSC-A) (Fig. S7A), then the removal of cellular debris due to the cell preparation was done by the threshold of population, based on both side scatter (SSC) and forward scatter (FSC) (Fig. S7B). Second, the settings of each photo-multiplying tube (PMT) for each fluorescent channel were done using non-transfected versus transfected cells (Fig. S7C-D). The ratio was calculated using the ratio of F_YFP_ (median fluorescence intensity)/F_CFP_ (median fluorescence intensity). A total of 1000–10000 events in triplicates were recorded. Data were analyzed using FlowJo™ software v10.8 (BD Biosciences, San Jose, CA, USA).

### Mitochondrial temperature

Mitochondrial temperature measurements were performed using the Mito-Thermo-Yellow dye (MTY) as previously described [5]. MTY fluorescence has been shown to be unaffected by changes in pH or membrane potential. Briefly, 48 h-transfected or non-transfected HEK293 cells were labeled with 100 nM MTY in a culture medium. After 30 min, cells were centrifugated at 1500 g for 5 min, and the pellet was washed in PBS. Then, cells were maintained as a concentrated pellet for 10 min at 37 °C to establish an anaerobiosis condition. The fluorescence (excitation 542 nm, emission 562 nm), the temperature of the medium in the cuvette, and the respiration of the intact cell suspension were simultaneously measured in a magnetically stirred, 37 °C-thermostated 1 mL-quartz cell, using a Xenius XC spectrofluorometer (SAFAS, Monaco). Potassium cyanide (KCN) was added to cells to inhibit mitochondrial respiration and thermogenesis when the maximal mitochondrial temperature was reached, i.e., when the MTY fluorescence was stable at its lowest level. The mitochondrial temperature was then calculated from the difference between the minimal and the maximal MTY fluorescence, obtained with mitochondrial heat production linked to respiration and its inhibition with KCN, respectively. Cell medium cooling was used to calibrate MTY fluorescence (Fig. 4A).

### Statistical analysis

Data are presented as median ± interquartile range. All statistical analyses were performed using GraphPad Prism 8 software. The non-parametric Mann–Whitney test was used to compare two groups. For multiple comparison, the Kruskal–Wallis test followed by Dunn’s post-hoc test or a two-way ANOVA followed by a Bonferroni post-hoc test were used when appropriate. Differences were considered significant when the p-value is less than 0,05.

## Supplementary Information

Below is the link to the electronic supplementary material.

Supplementary file1 (DOCX 721 KB)

Supplementary file2 (DOCX 570 KB)

## Data Availability

All data and materials presented in this article are available from the corresponding authors upon request.

## Abbreviations

CAP: Capsaicin
EHS: Exertional heat stroke
GTEx: Genotype-tissue expression
HEK: Human embryonic kidney
IMM: Inner mitochondrial membrane
MAMs: Mitochondria‐associated membranes
mitoTRPV1: Mitochondrial TRPV1
ML: Maximum likelihood
MTS: Mitochondrial targeting sequence
MTY: Mito-thermo-yellow
ORF: Open reading frame
pmTRPV1: Plasma membrane TRPV1
RTX: Resiniferatoxin
RYR: Ryanodin receptor
SERCA: Sarco-endoplasmic reticulum Ca^2+^-ATPase
TRPV: Transient receptor potential vanilloid
