# Supplementary material for: Identification of a human mitochondrial isoform of TRPV1: a thermostat regulating mitochondrial maximal temperature?
Source: Cell Mol Life Sci. 2026 Jul 23;83(1):339. doi: 10.1007/s00018-026-06360-5 (PMC13594010; doi:10.1007/s00018-026-06360-5)
Supplement: Supplementary file 1 [file 18_2026_6360_MOESM1_ESM.docx]

**Figure S1. Schematic representation of pmTRPV1 (top) and mitoTRPV1 (bottom) and the position of G684V amino acid change in both structures**

Ank: Ankyrin repeat domain; TM: Transmembrane Domain

**Figure S2. Phylogenetic prediction for the presence of an MTS in *TRPV1* gene in vertebrate species**

**A** Prediction score for the presence of MTS in TRPV1 gene from 198 vertebrate species. **B Phylogenetic tree based on the possible TRPV1 MTS protein sequences from the same species collected from NCBI and a home-made algorithm. A** and **B** Range pastel colors indicate the different clade for each species (Purple: Fish, Red: Amphibia, Orange: Reptile, Green: Bird, Blue: Mammal). Box colors represent the probability of the presence of an MTS predicted by MitoProt II. Color gradient from green (high probability p=1) to red (low probability p=0).

**Figure S3. Cellular fractionation of HEK293 cells expressing mitoTRPV1-GFP.**

Cell fractions correspond to cell lysate (Lysate), the crude cytoplasmic fraction (Cr.cyto), which is composed of the cytoplasm (cyto) and endoplasmic reticulum (ER) fractions, the crude mito fraction (Cr.mito), which is composed of the mitochondria (Mito) and mitochondrial associated membrane (MAMs) fractions. MitoTRPV1-GFP is detected using an anti-GFP antibody. VDAC and GRP75 antibodies are used to detect MAMs, TFAM antibodies to detect mitochondria and SERCA antibodies to detect the ER.

**Figure S4. Assessment of mitoTRPV1 pharmacological properties in mitoplasts**

**A** Representative mitoTRPV1 single-channel recordings present in mitoplasts isolated from transfected HEK293 cells, using a symmetric 150/150 mM isotonic KCl solution containing 2 mM Ca^2+^ at voltages ranging from -60 to +60 mV.

**B** The current-voltage (I-V) relationship based on single-channel recordings of mitoTRPV1 shown in **A**. Data were calculated based on experiments conducted in a symmetric 150/150 mM KCl isotonic solution containing 2 mM Ca^2+^, n=5.

**C** Open probability analysis of mitoTRPV1 at different voltages, based on the recording as in **B**, n=5.

**Figure S5. Ca^2+^ variations in cellular compartments from HEK293 expressing mitoTRPV1 or pmTRPV1.**

**A** Scatter plots representing the cell-by-cell maximal amplitude of RTX-response of normalized Fura-2 fluorescence ratio reflecting the cytosolic Ca^2+^ concentration ([Ca^2+^]_c_), in HEK293 cells expressing pmTRPV1 (green) or mitoTRPV1 (red) isoform and control cells (blue) stimulated with RTX (1 µM) in a Ca^2+^-free medium (CFB).

**B-C-D** Scatter plots of resting Ca^2+^ concentrations: in the cytoplasm measured with Fura-2 dye (b), in mitochondrial hot spot measured with N33D3cpv sensor (c), in mitochondria measured with 4mtD3cpv sensor (d) in CTRL cells (blue), mitoTRPV1 (red) and pmTRPV1 (green) expressing cells. **E** Top: Representative time traces of normalized 4mtD3cpv fluorescence ratio, reflecting the mitochondrial matrix Ca^2+^ concentration ([Ca^2+^]_matrix_), in HEK293 cells expressing pmTRPV1 (green) or mitoTRPV1 (red) isoform and control cells (blue) stimulated with RTX (1 µM). Bottom: scatter plots representing the corresponding cell-by-cell maximal amplitude of RTX-response (CTRL, n=92; pmTRPV1, n=141; mitoTRPV1, n=116). **F** Top: Representative time traces of normalized N33D3cpv fluorescence ratio, reflecting the mitochondrial Ca^2+^ hot spot concentration ([Ca^2+^]_hot spot_), in HEK293 cells expressing pmTRPV1 (green) or mitoTRPV1 (red) isoform and control cells (blue) stimulated with RTX (1 µM). Bottom: scatter plots representing the corresponding cell-by-cell maximal amplitude of RTX-response (CTRL, n=38; pmTRPV1, n=43; mitoTRPV1, n=18). Data are from at least three independent experiments. p-value correspondences *: p<0.05, **: p<0.01, ***: p<0.001, **** p<0.0001

**Figure S6. Impact of mitoTRPV1 expression on mitochondrial non-phosphoryalting and phosphorylating respiration rates in permeabilized HEK293 cells.**

**A** Representative trace of O_2_ consumption in permeabilized HEK293 cells. O_2_ concentration in blue and O_2_ flux in red. The addition of substrates and inhibitors are shown in black. Mitochondrial respiratory rates are measured after subsequent addition of 2.5 mM pyruvate and 5 mM malate (PM) (state II, complex I), 1.5 mM ADP (state III, complex I), then 5 mM glutamate (G) (state III, complex I), 10 mM succinate (S) (state III, complexes I andII), 2.5 µM rotenone (R) (state III, complex II), 4 µg/ml oligomycin (O) (state IV, complex II). Cell permeabilization and mitochondrial membrane integrity are checked by the subsequent addition of 1 µM FCCP (F) and 8 µM cytochrome c (C). Cytochrome c oxidase maximal respiration is tested by the addition of 4mM ascorbate (Asc) and 0.3 mM N,N,N'N'-tetramethyl-1,4-phenylenediamine (TMPD) followed by inhibition with 1 mM potassium cyanide (KCN) and 2 mM sodium azide (Azide). **B** Scatter plots of parameters corresponding to the different respiration states of CTRL (blue) or mitoTRPV1 expressing (red) cells.

**Figure S7. Gating parameters and strategy for mitochondrial ATP quantification using mitoATeam sensor** **and flow cytometry**

Description of the standard strategies for the assessment of singlets based on FSC-height (FSC-H) and FSC-area (FSC-A) and for gating cells based on forward scatter (FSC) and side scatter (SSC). To analyze transfected cells, further gating is applied to the singlet population based on mCherry (PE-Texas Red-A) and CFP/YFP(AmCyan-A/FITC-A) fluorescence intensities.
