# Supplementary material for: Identification of a human mitochondrial isoform of TRPV1: a thermostat regulating mitochondrial maximal temperature?
Source: Cell Mol Life Sci. 2026 Jul 23;83(1):339. doi: 10.1007/s00018-026-06360-5 (PMC13594010; doi:10.1007/s00018-026-06360-5)
Supplement: Supplementary file 2 [file 18_2026_6360_MOESM2_ESM.docx]

**Table S1. Table listing the primers and their sequence, used to clone wild-type or mutated mitoTRPV1 or pmTRPV1 with or without tags.**
